# Factors Related to Disclosure and Nondisclosure of Dietary Supplements in Primary Care, Integrative Medicine, and Naturopathic Medicine

**DOI:** 10.23937/2469-5793/1510109

**Published:** 2019-08-08

**Authors:** Jennifer R. Guzman, Debora A. Paterniti, Yihang Liu, Derjung M. Tarn

**Affiliations:** 1 Department of Anthropology, State University of New York at Geneseo, USA; 2 Department of Sociology, Sonoma State University, USA; 3 Departments of Internal Medicine and Sociology, University of California - Davis, USA; 4 United Healthcare Group, USA; 5 Department of Family Medicine, University of California – Los Angeles, USA

**Keywords:** Physician-patient relations, Communication, Dietary supplements, Patient disclosure, Qualitative research

## Abstract

**Background:**

Patients infrequently disclose use of dietary supplements to providers. Little is known about factors that motivate patients to disclose supplement use. The study aimed to identify reported factors motivating patients’ disclosure and nondisclosure of dietary supplement use and explore differences based on type of supplement and provider practice.

**Methods:**

Mixed methods study combining qualitative content analysis of semi-structured interviews with statistical analyses to assess differences in identified factors by provider practice type and supplement type. Seventy-eight English-speaking patients who reported taking 466 dietary supplements in the previous 30 days were recruited from primary care and Complementary and Alternative Medicine (CAM), and Integrative Medicine (IM) offices in Southern California.

**Results:**

We identified nine themes related to disclosure and nine related to nondisclosure of dietary supplement use. Major themes were features of the office visit, circumstances in patient health and medical care, and provider/patient characteristics. The most commonly raised theme promoting disclosure of supplement use was provider inquiry. Patients associate disclosure with having concerns about a supplement but also with annual physical exams and some routine topics of discussion, including self-care, lab results, and new medication prescriptions. Themes related to nondisclosure included lack of provider inquiry, features of the office visit, such as supplements being unrelated to the visit purpose, and patients’ convictions that supplements are safe or not important to discuss. Themes did not vary by supplement type. Primary care patients were more likely than CAM/IM patients to attribute nondisclosure to convictions that supplements were beneficial, not worth mentioning, or equivalent to food (p ≤ 0.001).

**Conclusions:**

When providers fail to ask directly about dietary supplement use, disclosure is often an impromptu decision that is driven by the content of provider-patient interactions. Ensuring disclosure of dietary supplement use to prevent potential drug-supplement interactions or adverse health outcomes likely requires consistent, proactive provider queries about supplement use.

## Introduction

In the United States, dietary supplements are a pervasive form of preventative, adjunctive, and alternative healthcare [1–10]. More than half of US adults use dietary supplements, among whom 12% report using five or more products [4]. Concurrent use with prescription medications is high [6,9,11]. Some supplements may provide health benefits [12–14]. However, supplement use can result in serious adverse health outcomes such as renal and liver injury [7,15–24], particularly for patients with pre-existing liver or kidney disease [7,19]. Studies have shown that while rare, the most serious supplement-drug interactions can result in renal and liver toxicity, cardiotoxicity, cardiovascular collapse, hypovolemic shock, and even death [21–23]. Because of such risks, the Food and Drug Administration (FDA) recommends that patients consult with a healthcare professional before using supplements [25]. However, most patients take dietary supplements on their own, and infrequently disclose their use to healthcare providers [7,26–37].

Numerous studies have identified factors associated with nondisclosure of supplement use to conventional medical providers. Patients are less likely to disclose if a provider does not ask about supplement use [29,36,38,39] or when they perceive time constraints [27,29,36]. Patients also report nondisclosure when they feel their provider is disinterested, unreceptive, or unknowledgeable about supplements [26,27,29,36,38,41], or may disapprove or respond negatively to supplement use [26,27,29,36,38,40,41]. Others do not disclose because they feel it is unimportant [27,36,38,39] or that they already have the information they need [27].

Comparatively less is known about the circumstances that *prompt* patient disclosure of dietary supplement use. Conventional physicians’ queries about supplement use are associated with disclosure [27]. And patients are more likely to disclose Complementary and Alternative Medicine (CAM) use, including dietary supplements, if a professional recommended the supplement [42], if it was used to treat a medical condition [42], or if patients believed the provider was knowledgeable or open-minded about CAM [26,30,38]. Disclosure may also be motivated by the belief that disclosure is important for safety or to help others with the same condition [30].

The research literature lacks information about the specific reasons patients disclose dietary supplement use during a given office visit, and whether the types of supplements patients are taking influence whether they disclose their use. Patient reasons for disclosing or not disclosing to CAM/Integrative Medicine (IM) practitioners have not been studied at all. This study aims to (1) Explore patient reasons for disclosure and nondisclosure of dietary supplement use during a particular outpatient office visit, as well as generally, and (2) Determine whether those reasons vary by supplement type or provider specialty (conventional primary care versus CAM/IM doctors).

## Methods

### Design

The data are from a larger study concerning provider-patient communication about dietary supplements [43]. Between November 2011 and May 2013, 603 patients were recruited from 61 participating provider waiting rooms (32 primary care physicians from academic, community health, and managed care practices, 14 integrative medicine physicians from academic and private practices, and 15 CAM providers - 5 naturopaths, 5 acupuncturists, 5 chiropractors). Eligible patients were English or Spanish-speaking, at least 18 years old, and available to participate in a follow-up interview within one week of an audio-recorded office visit. Participants completed surveys about demographics and supplement use.

Among 477 of the 603 patients who reported taking dietary supplements in the past 30 days, 126 were purposively sampled for interviews based on practice type and supplement disclosure during their office visit. Eight of the 126 patients could not be reached, and one decided not to participate. Our analyses focused on 78 English-language interviews with patients of primary care and IM physicians and naturopathic physicians who held Naturopathic Doctor (ND) degrees. Since these providers all practice primary care [44–47], they may be more likely to solicit complete medication and supplement information than chiropractors and acupuncturists, who most frequently consult about more focused complaints [48–50]. Patients received a $25 gift card upon interview completion. The Institutional Review Boards of the University of California, Los Angeles and Kaiser Permanente approved the study protocol.

Trained and experienced interviewers--a medical anthropologist (JRG) and a medical linguist--conducted all patient interviews via telephone. Interviewers used one of two semi-structured interview guides [51] containing slightly different questions for interviewees who did and did not disclose supplement use during the recorded visit. Dietary supplements were defined for interviewees as “vitamins, minerals, and herbal supplements” [52]. We asked interviewees who disclosed supplement use about the circumstances and content of their discussions and their reactions to them (Table 1). Interviewees reporting nondisclosure were asked why, and what circumstances could have prompted disclosure. We asked all interviewees who should initiate discussions and when disclosure is necessary/unnecessary. We used follow-up probes [53] for clarification as needed. Interviews lasted an average of 29.7 (SD = 9.2) minutes and were digitally recorded and transcribed verbatim.

### Categorization of supplements

Every supplement mentioned in the interviews was categorized into: (1) Supplements commonly recommended by primary care providers (i.e. calcium, vitamin D, fish oil/omega-3, folic acid, and glucosamine with/without chondroitin or methylsulfonylmethane), (2) Multivitamins/multiminerals, (3) All other vitamins/minerals, (4) Non-vitamins, non-minerals (NVNM) and (5) General, a category to capture comments interviewees made about disclosure and nondisclosure of supplements, regardless of type. The fifth category included cases where interviewees talked about circumstances in which they considered disclosure necessary or unnecessary. Our determination of disclosure or nondisclosure of each supplement was based on interviewee report during the interview.

### Qualitative analysis

Each interview transcript was verified for accuracy prior to analysis. Two coders, a practicing primary care physician with research expertise in provider-patient communication (DMT) and a medical anthropologist (JRG), conducted inductive content analysis [54] on a subset of interviews, (1) Identifying themes related to disclosure or nondisclosure of each supplement interviewees were taking and of supplements in general and (2) Categorizing themes and subthemes. A medical sociologist with expertise in qualitative methods (DAP) reviewed a partially overlapping set of transcripts and an additional set of transcripts for validity of coding. They also worked together to finalize the coding scheme and develop a codebook defining each theme.

One investigator (JRG) coded all interviews, one of the other two investigators double-coded 20 interviews (26%), and all three investigators coded six interviews (8%). Multiple investigators coded at least 10% of the interviews from each practice type. Throughout the coding process, the investigators iteratively reviewed the coding scheme and incorporated new themes until saturation was reached [55]. Coding discrepancies between auditors were discussed and resolved by group consensus with consideration of the decision’s relevance to clinical practice. Atlas.ti 7 (Scientific Software Development, Berlin, Germany) was employed for systematically storing, coding, retrieving, and reviewing coded data.

### Statistical analysis

All statistical analyses were conducted using SAS, version 9.1. Summary statistics, including means and percentages, were calculated to describe participants’ characteristics. We used generalized linear mixed models to examine the relationship of themes identified through our qualitative analysis of the interviews on patient disclosure or nondisclosure of individual dietary supplements. Generalized linear mixed models were selected because they allow us to control for the possible correlation of behaviors within patients. For this analysis, any patient mention of a theme pertaining to disclosure or nondisclosure of dietary supplement use counted as fulfillment of the theme. We also used generalized linear mixed models to assess differences in mentions of themes related to dietary supplement disclosures and nondisclosures by practice types (conventional primary care versus integrative/naturopathic) and supplement type (supplements commonly recommended by primary care providers; multivitamins and multiminerals; all other vitamins and minerals; and NVNM).

## Results

### Patient and supplement characteristics

Of the 78 patients in the study, 45 were primary care, 23 were IM, and 10 were naturopathic patients. Patients were predominantly white, female, and highly educated. Compared to integrative and naturopathic patients, primary care patients had significantly lower educational achievement (*p* = 0.02) and were younger and more ethnically diverse (though these differences were not statistically significant) (Table 2). Forty participants (51.2%) reported disclosing at least one supplement to their provider during their office visit. Patients discussed between one and 18 supplements during their interviews (mean 6.0, SD = 3.7), and a total of 466 supplements across all interviews (39 multivitamin/multi-minerals, 101 supplements commonly recommended by primary care providers, 104 other vitamin/mineral supplements, and 222 NVNM). Of these, interviewees reported disclosing 156 (33%) to their provider.

### Themes promoting disclosure and nondisclosure

We identified twelve major themes related to disclosure/nondisclosure (Table 3), which spanned three domains: features of the office visit, patient health and medical care circumstances, and provider and patient characteristics. Interviewees mentioned six themes in relation to both disclosure and nondisclosure three in relation to disclosure only, and three in relation to nondisclosure only.

#### Themes promoting disclosure:

The themes related to disclosure that emerged most frequently were related to features of the office visit. Many interviewees attributed their disclosure of supplement use to *interaction during the visit*, specifically provider inquiries. For example, “Doctor [surname] asked what supplements I take, and I just mentioned that I take that for heart and skin health” (688). Unprompted disclosures were most often attributed to *discussion topics* pertinent to supplement use, e.g. patient self-care and laboratory results. As one interviewee explained, “When he was asking me about sleep, I immediately mentioned that I had been using the melatonin” (234). Occasionally, interviewees associated *visit characteristics* with disclosure, i.e. pre-surgery visits, physical examinations, or new patient visits.

Many interviewees associated aspects of patient health and medical care circumstances with disclosure. Having *concerns about a supplement*, including perceived or feared side effects and questions about safety, commonly emerged. Few reported actual concerns, but many said they would disclose if they had them, or to get advice. Some interviewees said patients should disclose when taking unusual or multiple supplements, mega-dosing, or changing a supplement regimen (*circumstances of supplement use*). Many interviewees believed that disclosure was important under *medication-related* circumstances; for example, “It needs to be brought up any time that there’s going to be a prescription that’s not something you’ve taken before” (172). A minority of interviewees said disclosure is necessary when patients have *medical conditions* (“I have a lot of medical problems, so I always, every time I see her, I just kind of put her up to date” (838)).

Finally, interviewees occasionally cited provider and patient characteristics. Several noted that *provider characteristics* like expertise/knowledgeability or receptiveness to discussing supplements gave them confidence to disclose. A few interviewees conveyed personal *convictions* about the importance of disclosure.

#### Themes promoting nondisclosure:

Most interviewees invoked multiple themes concerning nondisclosure. Numerous interviewees attributed nondisclosure to *interaction during the visit*, specifically the topic not coming up. Frequently mentioned *visit characteristics* associated with nondisclosure include when supplements are unrelated to the visit’s purpose, when there are competing demands or time constraints, or when they thought the visit type did not warrant disclosure (i.e. follow-up and acute care visits). Nondisclosure was also attributed to *organizational or procedural factors*: when the patient is unprepared to discuss supplement use or believes the provider already knows about their supplement use.

Nondisclosure was often associated with the *circumstances of supplement use*: sporadic use, long duration of use, or use of a supplement considered to be safe. One patient taking Coenzyme Q10 noted “I thought that’s very safe to take, and so I didn’t discuss it with my doctor” (565). For others, good health or fitness (*medical condition*) made discussion unnecessary (e.g., “I wouldn’t really feel the need to tell him because I’m in relatively good shape” (151)).

According to some interviewees, *provider characteristics* militated against disclosure: lack of relevant expertise, unreceptiveness to supplement discussions, and prejudice against supplements. Others attributed nondisclosure to *convictions* about supplements: they aren’t worth mentioning, are beneficial/commonplace, or are equivalent to food nutrition. One patient explained, “Usually I talk to him about everything. But just being a vitamin, I didn’t feel it [was] necessary” (156). Some interviewees expressed *confidence* in their own knowledge about supplements. Finally, some reported that supplements *did not cross their mind* during the office visit (“It’s not a decision not to bring them up. It’s more just not really thinking about it” (560)).

### Statistical differences in disclosure and nondisclo- sure of supplements

Statistical analyses revealed that interviewees reported disclosing supplement use less frequently to primary care physicians (13% of supplements) than to integrative/naturopathic practitioners (45%) (p < 0.01). Five of the nine themes related to disclosure were significantly associated with reported disclosure (Table 4): Interaction during the visit (p < 0.001), discussion topic (p < 0.001), patient concerns about supplement (p < 0.01), medication related (p < 0.001), and provider characteristics (p < 0.001). Visit characteristics, circumstances of supplement use, and medical conditions were not significantly related to reported disclosure. Six themes related to nondisclosure of supplement use were significantly associated with reported nondisclosure: interaction during the visit (p ≤ 0.01), visit characteristics (p < 0.001), organizational and procedural factors (p < 0.001), circumstances of supplement use (p < 0.01), provider characteristics (p = 0.04), patient convictions (p ≤ 0.001), and did not cross mind (p ≤ 0.001). Medical condition and patient confidence were not significantly related to reported nondisclosure.

There were no differences in the frequency with which patients of primary care and integrative/naturopathic providers mentioned themes promoting dietary supplement disclosure. However, primary care patients were more likely than integrative/naturopathic patients to raise several themes associated with nondisclosure of specific supplements (data not shown): circumstances of supplement use (SE 1.36, p = 0.02), medical condition (SE 5.81, p = 0.03), patient convictions (SE 1.84, p ≤ 0.001), and patient confidence (SE 8.13, p = 0.03). There were no differences in how often patients raised themes about disclosure and nondisclosure based on supplement type.

## Discussion

This study expands existing knowledge about patients’ motivations for disclosing dietary supplement use to primary care, integrative medicine, and naturopathic providers. Findings reveal several factors promoting disclosure that have not been previously identified in the literature. Previous research suggests that a provider’s “communication style [30]” (i.e. receptive/non-judgmental versus discouraging/critical) is a moderating factor of disclosure. However, this study suggests that disclosure is often an impromptu decision driven by unfolding interactional opportunities. Nondisclosure may be the default practice for most patients, who typically fail to think about their supplement use during visits and who disclose only when they have a specific concern or a certain threshold of relevance to the topic-at-hand is reached. Patients associate disclosure with out-of-the-ordinary circumstances, such as having concerns about a supplement they are taking or when mega-dosing. However, patients also associate disclosure with certain more routine topics of discussion, including talk about health problems the patient is treating with supplements and lab results they believe may be influenced by supplement use. Patients also view disclosure as warranted when a new medication is being prescribed. Some types of office visits are viewed as more appropriate occasions for disclosing dietary supplement use than others, specifically annual physicals and new patient appointments versus acute care and follow-up visits.

Physician awareness of the circumstances patients associate with disclosure may enable them to better foster disclosure and discussion of supplement use. If a patient discloses use of a dietary supplement during a discussion of laboratory results, for example, the physician may be able to use the opportunity as an interactional wedge to ask about other supplement use, producing a sort of domino effect for disclosure. To take advantage of factors that promote disclosure, provider education to increase inquiries about supplement use, which other studies have recommended [26,27,29,33,34,38], could train doctors to target optimal occasions, as well as encouraging the sort of receptive communication style that facilitates patient trust and willingness to disclose. Taking advantage of interactional opportunities to pursue non-judgmental supplement discussions may serve as a way for doctors to dispel patient perceptions that providers are not interested in talking about dietary supplements [26,27,29,38,41] and to convey why disclosure is vital for ensuring patient health by mitigating potential adverse interactions and health outcomes, particularly in patients with pre-existing medical conditions [7,19].

Several factors associated with nondisclosure were more prevalent in primary care than integrative/naturopathic settings, suggesting that conventional primary care doctors face greater challenges to learning what supplements their patients are taking. These challenges include several patient convictions: that supplements are beneficial, commonplace, or equivalent to nutrition from food and that these characteristics make disclosure unnecessary. Other factors associated with nondisclosure that may disproportionately affect primary care practitioners include patients’ confidence in their own good health and their knowledge about the supplements they are taking. Across practice types, patients do not appear to distinguish between products that may require consultation with a healthcare professional (e.g., herbal NVNM supplements, which may interact with prescription medications) and those that do not. Possible interventions to promote disclosure of dietary supplement use may include patient education about the risks of supplement-medication interactions and the importance of disclosing supplement use. Such interventions could be targeted to patients who take prescription medications or have chronic conditions that make supplement use particularly risky [7,19,56–58]. Interventions targeted to providers could also emphasize the importance of inquiring about supplement use with these kinds of patients during chronic care visits and visits when changes are made to patient medication regimens.

The findings of this study are limited by the study population. The study sample was small and non-random, recruited exclusively in urban Southern California and overrepresented individuals with high educational achievement and non-Hispanic whites, who may not be representative of all dietary supplement users. Because use of dietary supplements varies across racial/ethnic groups, and disclosure rates for minorities are very low [4,32,34,42,59–62], further study of factors related to disclosure in these populations is warranted. Findings regarding disclosure are also limited because they rely on patient recall. Study of office visit recordings and transcripts is warranted to identify patterns in actual disclosure and nondisclosure.

In conclusion, the findings of this study suggest that promoting effective discussions about supplement use between patients and providers during office visits requires more than eliminating obstacles to disclosure and likely requires a proactive stance on the part of providers to inquire about supplement use and to pursue discussions when patients raise the topic. Given the typicality of nondisclosure, asking direct questions about a patient’s supplement use may be the most reliable way for providers to learn what a patient is taking. Posing questions in the contexts that patients associate with disclosure may help to maximize their effectiveness, and providers may need to address a range of patient convictions that militate against disclosure. Initiating discussion about supplement use may align with patient expectations about dietary supplement discussions [63], improve patient satisfaction [64,65] and provider-patient relations [66], and set a positive precedent for disclosure in the future.

## Figures and Tables

**Table 1:** Selected guiding questions for interview.

If you talked about the supplements you are taking during your last visit:
What prompted the discussion?
What was going on in your mind when you mentioned the supplement(s)?
What kept you from mentioning other supplements you are taking during your last visit?
If you did *not* talk about supplements:
Why did you not bring them up?
Is there anything that could have prompted you to discuss them?
Under what conditions do you feel it is necessary to discuss dietary supplement use?
Under what conditions do you feel it is *not* necessary to discuss dietary supplement use?

**Table 2:** Interviewee characteristics.

Characteristics	Primary care patients n = 45	Integrative/naturopathic patients n = 33	*p*-value
Female, frequency (%)	28 (62.2)	25 (75.6)	0.20
Age in years, mean (standard deviation)	48.4 (18.2)	53.8 (13.7)	0.15
Race/ethnicity, frequency (%)			0.08
White	17 (37.8)	22 (66.7)	
Hispanic	11 (24.4)	2 (6.1)	
Black	7 (15.6)	4 (12.1)	
Asian	4 (8.9)	1 (3.0)	
Multiracial/Other	6 (13.3)	4 (12.1)	
Highest level of education, frequency (%)			0.02
High school or less	2 (4.4)	0 (0.0)	
Some college	19 (42.2)	6 (18.2)	
College graduate	24 (53.3)	27 (81.8)	

**Table 3:** Themes related to disclosure and nondisclosure of dietary supplement use during office visits^1^.

Domains	Themes	Disclosure examples	Nondisclosure examples
Features of the office visit	Interaction during the visit	**Provider inquiry about supplement use** (157): He asked me what supplements I was taking. **Provider inquiry about what a patient is taking** (705): She asked me what I take daily, and I told her. (671): They always want to know what you are taking besides your medication.	**Provider didn’t ask about supplement use** (187): He didn’t specifically ask. (688): Any time they don’t ask [disclosure is unnecessary]. **Topic didn’t come up** (198): It just never comes up. I mean, you’re talking about a subject that just never, never, never comes up. (448): It [supplements] just didn’t come up.
	Discussion topic	**Self-care** (691): I had not been feeling well, and I said that I had used my herbal remedies, which was Vitamin C and Echinacea. (708): I mentioned that I was taking these [supplements] in addition to staying away from various foods. **Lab results** (802): We were itemizing the chem panel… and I started telling her, “Well, I take – I already take a multi.” (560): I was taking a supplement called Coenzyme-A…and it really, really skewed one of my lab results…and I mentioned, “These are what I’m taking”.	
	Visit characteristics	**Before surgery/procedure** (172): Before going into surgery, always discuss your supplements. (705): If someone’s going to do a brain cat scan on me tomorrow, I think they should know what I have ingested that morning. **Type of office visit** (418): I was going in for a physical and…I let him know I’m taking some Mass XXX. (455): If it’s an establishment visit, you definitely want to let your doctor know everything you’re taking.	**Supplements unrelated to purpose of visit** (672): I really wasn’t there to talk to him about supplements. (682): I went to see the doctor about something else. **Competing demands during visit** (151): I was more interested in addressing things that were probably way more important. (557): There are other things to discuss, and that doesn’t really seem pertinent to any of the problems. **Type of office visit** (424): This was more kind of a follow up visit. There was no reason for it to come up. (363): [Because] it wasn’t a general physical.
	Organizational and procedural factors		**Not on list to discuss** (329): [Supplement use] wasn’t on my list of things to talk about. **Provider already knows about supplement** (565): [The doctor] already knows that I’m taking all the vitamins and everything because … I usually tell these things to the nurse. (511): It’s in my summary, and I do not think I need to talk about.
Patient health and medical care	Concerns about supplement	**Concerns about side effects** (560): If you’re taking them and not feeling well…you need to bring it up. (811): I thought maybe Vitamin C was too much, that’s why it is giving urinating (sic) too often. **Safety concerns** (187): I just wanted to make sure [fish oil] was safe. (867): I wanted to know if it was safe or not. **Request advice from provider** (329): To ask…whether or not a couple of these supplements I was taking them right. (857): It’s important for me to talk to her about my supplement use because…I want her input.	
	Circumstances of supplement use	**Taking unusual/multiple/mega-dose supplements** (363): If I were to go on high potency regimen…then I think I should talk to the doctor. (560): If you thought that you were taking something that may be kind of controversial … you should bring it up. **Change in supplement use** (591): I give her information every time I change my supplement list. (618): Every time I have a visit with a doctor, I talk about supplements. Or I talk about any additional supplements.	**Sporadic use** (362) If you’re very sporadic about taking supplements, it’s probably not necessary to bother the doctor with that. (110): Light use…is why I didn’t really bring it up – you know, a handful of Vitamin D, fish oils, Vitamin C, that kind of thing sporadically. **Long duration of use** (329): I…didn’t bring it up because I’ve been taking them regularly for so long. **Supplement considered safe** (465): I don’t see it as something that would harm me. (565): I thought [Co-Q10]’s very safe to take, and so I didn’t discuss it with my doctor.
	Medication related	**Taking prescription medication** (329): If you’re on a regular medication, you want to make sure your doctor knows that you’re also on these other things. **Prescribed new medication** (110): I would have discussed it if I were being prescribed a new medicine. (158): What made me talk about it [was] … I was kind of concerned about what she was giving me for my acid reflux…would it counteract the enzymes.	
	Medical condition	**Having a medical condition** (228): Anybody that has any health challenges should [disclose]. (820): I think with psychiatric conditions it is important.	**Good health/fitness** (363): If my health is stable and good…I don’t think it has to be brought up. (557): Because we’re healthy. (560): If I go in and my labs are perfect, and everything’s good, I don’t feel the need to discuss it.
Provider and patient characteristics	Provider characteristics	**Doctor expertise/knowledge** (835): She is a naturopath…so it was a built-in topic. (591): I want them all to know [what I’m taking] because they’re very knowledgeable. **Doctor receptiveness** (122): It’s a very open-door type of situation where I could call her and tell her … I started taking something. (749): If my practitioner is open to that I would talk about it.	**Doctor lack of knowledge** (455): I’m sure it wouldn’t hurt [to disclose], but…I would doubt that she would know what they are. **Doctor unreceptiveness/disinterest** (157): I would never discuss it because they just don’t want to hear about it. (151): I never would even think that a Western doctor would really be that interested. **Prejudice against supplements** (857) I’m probably not going to say I’ve tried to treat this with D-Mannose, or probiotics… because I just feel like there’s…a bias against it.
	Patient convictions	**Important to disclose supplements** (688): I think a doctor should know everything that you consume regularly. (705): I feel pretty strongly that she needs to have an up-to-date list of what I’m taking.	**Not worth mentioning** (234): It’s just something that I don’t think is worth mentioning. **Supplements are beneficial/commonplace** (816): I didn’t feel like I needed to bring them up because…vitamins and herbs are beneficial. (867): The Emergen-C, I think that’s just a good drink… so I didn’t think anything of it. **Supplements are equivalent to food** (648): Because I figured…everything we eat has vitamins in it anyway. (110): Because … Vitamin C is in most fruit, if not all, I think. Vitamin D we get from the sun. Fish oils you get when you eat fish.
	Patient confidence		**Confidence in own knowledge** (820): I…am pretty savvy with what I ingest, so I didn’t think [mentioning] it was worthwhile. (560): I’ve done quite a bit of research in regard to the affect they have on the body… so I’m pretty well educated.
	Did not cross mind		**Did not cross mind** (672): It just didn’t occur to me.

1 Numbers in parentheses refer to the interviewee ID number.

**Table 4:** Generalized Linear Mixed Models for relationship of identified themes on supplement disclosure and nondisclosure (n = 466 supplements).

	Estimate for relationship to supplement disclosure*	Standard Error	p-value
**Themes related to disclosure**			
Interaction during visit	1.89	0.45	< 0.001
Discussion topic	2.63	0.43	< 0.001
Visit characteristics	− 0.65	1.06	0.54
Concerns about supplement	1.22	0.47	< 0.01
Circumstances of supplement use	− 0.09	1.00	0.93
Medication related	2.79	0.79	< 0.001
Medical condition	− 1.04	1.47	0.48
Provider characteristics	2.28	0.66	< 0.001
Patient convictions			
**Themes related to nondisclosure**			
Interaction during visit	− 3.48	1.07	< 0.01
Visit characteristics	− 3.94	0.74	< 0.001
Organizational and procedural factors	− 3.63	0.67	< 0.001
Circumstances of supplement use	− 1.85	0.59	< 0.01
Medical condition			
Provider characteristics	− 1.53	0.75	0.04
Patient convictions	− 2.51	0.69	< 0.001
Patient confidence	− 0.78	0.63	0.02
Did not cross mind	− 4.72	1.19	< 0.001

* reference group = supplement nondisclosure.
